# Peroxisomes in Aging: Guardians of Cellular Resilience and Function

**DOI:** 10.3390/cells15030254

**Published:** 2026-01-28

**Authors:** Artuur Vercaemst, Mingming Zhao, Ruizhi Chai, Celien Lismont, Marc Fransen

**Affiliations:** Laboratory of Peroxisome Biology and Intracellular Communication, Department of Cellular and Molecular Medicine, KU Leuven, 3000 Leuven, Belgium; artuur.vercaemst@kuleuven.be (A.V.); mingming.zhao@kuleuven.be (M.Z.); ruizhi.chai@kuleuven.be (R.C.); celien.lismont@kuleuven.be (C.L.)

**Keywords:** aging, catalase, interorganelle crosstalk, lipid metabolism, metabolic disorders, neurodegeneration, peroxisomes, pexophagy, reactive oxygen species, therapeutic interventions

## Abstract

**Highlights:**

**What are the main findings?**

- Peroxisomal functions decline with age, impairing protein import, lipid metabolism, redox balance, and organelle quality control.
- Peroxisomes act as central signaling hubs, and their dysfunction propagates stress to mitochondria, the endoplasmic reticulum, and lysosomes.

**What are the implications of the main findings?**

- Peroxisomal homeostasis supports healthy aging by contributing to cellular resilience and metabolic stability.
- Peroxisomal pathways are emerging as therapeutic targets for age-related diseases and functional decline.

**Abstract:**

Peroxisomes are multifunctional organelles that play essential roles in lipid metabolism, redox regulation, and cellular signaling. An expanding body of evidence implicates peroxisomal dysfunction as a key contributor to aging and age-related diseases. Aging is accompanied by progressive declines in key peroxisomal functions, including catalase activity, fatty acid β-oxidation, plasmalogen biosynthesis, and the metabolism of bile acids and docosahexaenoic acid, resulting in increased oxidative stress, lipid dysregulation, and alterations in membrane composition. Impaired pexophagy further exacerbates these defects by allowing the accumulation of damaged peroxisomes and compromising cellular homeostasis. Through extensive metabolic and signaling crosstalk with mitochondria, the endoplasmic reticulum, and lysosomes, peroxisomal dysfunction can propagate oxidative and metabolic disturbances throughout the cell. In addition, peroxisome-derived signaling molecules, such as hydrogen peroxide and bioactive lipids, link peroxisomal activity to cellular stress responses and organismal metabolic homeostasis. We propose that aging-associated impairments in peroxisomal protein import, redox regulation, and selective turnover progressively shift peroxisomes from adaptive metabolic signaling hubs toward sources of chronic oxidative and lipid stress. In this context, current studies highlight peroxisomal homeostasis as a potential determinant of healthy aging and point to peroxisomal pathways as emerging targets for intervention in age-related disease.

## 1. Introduction

Aging is a natural process that progressively compromises human health by affecting multiple biological and physiological systems, including metabolism, organ function, cognition, and immune competence, ultimately reducing mobility, independence, and quality of life [1,2,3,4]. Although these changes increase vulnerability to chronic and neurodegenerative diseases, lifestyle factors such as regular physical activity, balanced nutrition, intermittent fasting, cognitive stimulation, and social engagement can delay or attenuate many age-related declines, highlighting the potential for interventions that promote healthy aging [5,6]. At the cellular level, aging reflects the cumulative impact of intrinsic processes—including genomic instability, telomere shortening, organelle dysfunction, oxidative stress, and cellular senescence—that disrupt tissue homeostasis and drive disease susceptibility [7]. Understanding how these mechanisms intersect is essential for elucidating the biological basis of aging. In this review, we synthesize emerging evidence that implicates age-associated deterioration of peroxisomal homeostasis—characterized by impaired protein import, redox imbalance, and defective turnover—as a contributor to metabolic dysfunction and age-related disease, and we discuss how maintaining peroxisomal integrity may support cellular resilience during aging.

## 2. Peroxisomal Functions and Their Role in Aging

Peroxisomes are single-membrane organelles found in nearly all mammalian cells [8], with the exception of mature erythrocytes [9], spermatozoa [10], and lens fibers [11]. They are essential for lipid metabolism, redox balance, and cellular signaling [12,13]. Peroxisomes perform key metabolic processes, including the α- and β-oxidation of various lipophilic carboxylic acids [14], the biosynthesis of plasmalogens [15] and docosahexaenoic acid (DHA) [16], the production and detoxification of hydrogen peroxide (H_2_O_2_) via flavin-containing oxidases and catalase, respectively [17], and the breakdown of xenobiotic acyl compounds [18]. Age-related declines in peroxisomal function result in the accumulation of very-long-chain and branched-chain fatty acids, a reduction in plasmalogens, and increased oxidative stress, contributing to metabolic dysfunction and redox imbalance, as observed in Alzheimer’s disease patients [19] and a corresponding mouse model [20]. Because many of these metabolic and redox processes are closely shared with other organelles, including mitochondria [21], peroxisomal decline is expected to have broader consequences for cellular homeostasis.

Mitochondrial function is essential for maintaining health, and its progressive decline is a hallmark of aging [7]. Many cellular processes that preserve mitochondrial integrity—such as lipid metabolism and reactive oxygen species (ROS) homeostasis—rely on close cooperation with peroxisomes [22,23]. Despite this critical interplay, peroxisomes are often overlooked in mitochondria-centered theories of aging. They support mitochondrial performance by regulating lipid metabolism and controlling the levels of signaling molecules, including H_2_O_2_ and bioactive lipids such as DHA-derived mediators and neuroprotective lipids [16,17,24,25], which feed into cellular signaling networks to modulate stress responses and enhance resilience. Through dynamic interactions with mitochondria and other organelles—including the endoplasmic reticulum (ER), lysosomes, and lipid droplets [22,26]—peroxisomes coordinate metabolic and signaling processes that sustain organismal health. Both inherited defects and age-related functional decline can disrupt these interorganellar networks, underscoring peroxisomes as crucial, yet often underappreciated, regulators of aging and age-related diseases [27,28,29,30].

## 3. Aging-Associated Peroxisomal Dysfunction

**Figure 1:**
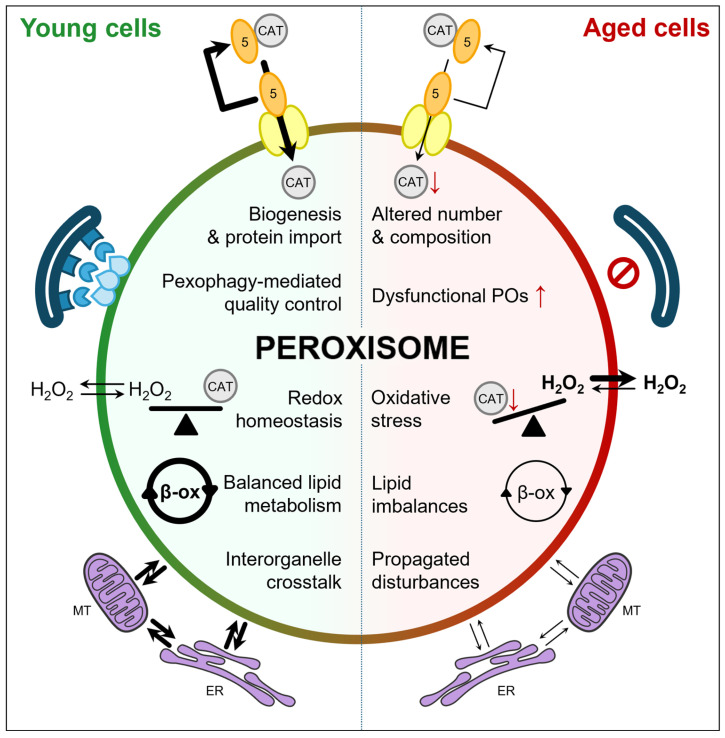
Age-associated decline in peroxisome homeostasis and function. Aging induces multifactorial alterations in peroxisome (PO) biology, including reduced peroxisome biogenesis and protein import, dysregulated organelle turnover via pexophagy, increased oxidative stress, and impaired lipid metabolic functions (depicted as β-ox), such as fatty acid β-oxidation, plasmalogen biosynthesis, bile acid metabolism, and docosahexaenoic synthesis. Collectively, these changes lead to age-related alterations in peroxisome number and composition, thereby promoting oxidative stress, lipid imbalance, and disrupted interorganelle communication. The PEX5 (5) docking/translocation machinery is shown in yellow, and the autophagic machinery involved in pexophagy is depicted in blue. ↑ indicates an increase; ↓ indicates a decrease; thicker arrows represent stronger effects; and the red circular regulatory symbol indicates reduced pexophagy. CAT, catalase; ER, endoplasmic reticulum; MT, mitochondria.

Aging has a pronounced impact on peroxisomes, influencing their abundance, enzymatic composition, and overall functional capacity in a cell- and tissue-specific manner [31,32,33,34]. These age-related changes result from multiple factors, including reduced peroxisome biogenesis, altered peroxisomal protein expression, impaired protein import, and modified peroxisome turnover. Collectively, these changes drive the progressive decline of peroxisomal function with age, which is examined in detail in the following sections focusing on biogenesis, protein import, quality control, H_2_O_2_ handling, and lipid metabolism (Figure 1).

### 3.1. Peroxisome Biogenesis and Protein Import

New peroxisomes are generated either de novo from pre-peroxisomal vesicles originating from the ER or mitochondria; or through the growth and division of existing organelles [35,36,37]. Their assembly and maturation are orchestrated by peroxins (PEX), which coordinate vesicle formation as well as the import of membrane and matrix proteins [38]. PEX16, a peroxisomal membrane protein (PMP), is required for ER-derived vesicle formation [35], whereas mitochondria-derived vesicles involve PEX3 and PEX14 [39,40]. PEX19 functions as a cytosolic chaperone and import factor for PMPs [41], whereas PEX5 and PEX7 act as cycling receptors for matrix proteins containing C-terminal (PTS1) or N-terminal (PTS2) peroxisomal targeting signals, respectively [42]. PEX5-cargo complexes dock at PEX13 and PEX14, allowing cargo translocation followed by PEX5 recycling [42]. Recycling occurs in two steps: first, PEX5 is monoubiquitinated on a conserved and redox-sensitive cysteine (Cys11 in humans) and retrotranslocated via the PEX2-PEX10-PEX12 ubiquitin ligase complex, which facilitates PEX5 unfolding and cargo release [43,44]; second, the PEX1-PEX6 ATPase, anchored to the membrane by PEX26, extracts PEX5 from the membrane in a process analogous to ER-associated degradation [45]. Mature peroxisomes proliferate through elongation, constriction, and fission, a process regulated by proteins such as PEX11α/β, fission protein 1, and dynamin-like protein 1 [46].

It is well established that cellular aging, a process associated with increased oxidative stress, affects PEX5 functionality [32,34,47,48,49]. Aging and oxidative stress impair PEX5 recycling, leading to its accumulation on the peroxisomal membrane and reducing the import of matrix proteins, particularly those with weak PTS1 signals [50]. A prominent example is catalase, which is retained in the cytosol under oxidative stress; together with the redox-regulated activity of PEX5, this serves as a cellular defense mechanism against oxidative insults originating outside the peroxisome [50] (Figure 1). Furthermore, both cellular and organismal aging have been shown to downregulate numerous peroxisomal proteins, including key factors required for peroxisome biogenesis. This includes PEX3 and PEX5 in *Caenorhabditis elegans* [48]; PEX5 in aged *Drosophila* oenocytes and intestinal stem cells, as well as in aged mouse intestinal stem cells [49,51]; and PEX2, PEX5, PEX10, PEX12, and PEX19 in humans [52]. As a result, both peroxisome biogenesis and enzymatic composition are altered with age, a pattern confirmed in human B-cells [34] and in subproteomic analyses of aged mouse liver and kidney peroxisomes [33]. Collectively, these findings demonstrate that peroxisome homeostasis—encompassing both quantity and functionality—is compromised during aging.

### 3.2. Peroxisomal Quality Control

Peroxisome homeostasis is maintained by a dynamic balance between organelle biogenesis and selective degradation through pexophagy, a specialized form of autophagy. Like other selective autophagy pathways, such as mitophagy, pexophagy can proceed via ubiquitin-dependent or ubiquitin-independent mechanisms to eliminate damaged or excess peroxisomes, thereby supporting metabolic flexibility. The molecular machinery underlying pexophagy has been reviewed in detail elsewhere [53,54,55].

Aging profoundly impairs autophagy [56], characterized by chronic mTOR activation [57], reduced AMPK and SIRT1-signaling [58,59], decreased expression and activity of core autophagy-related proteins [60], and increased oxidative stress impairing genomic instability [61], which together compromise autophagic flux and lysosomal function. Although direct longitudinal studies of pexophagy during organismal aging are limited [62,63,64], evidence from autophagy-deficient models [65] and senescent cells [66] suggests that pexophagy is similarly disrupted, leading to the accumulation of dysfunctional peroxisomes and heightened cellular stress (Figure 1).

### 3.3. Peroxisomal H_2_O_2_ Metabolism

Peroxisomes generate considerable amounts of H_2_O_2_ through the activity of flavin-containing oxidases, which participate in the β-oxidation of very-long-chain fatty acids (VLCFAs), pristanic acid, poly-unsaturated fatty acids (PUFAs), and bile acid intermediates, as well as the metabolism of D-amino acids (e.g., D-serine and D-aspartate), L-hydroxy acids (e.g., glycolate), polyamines (e.g., N^1^-acetylspermine), and L-pipecolic acid [67]. Catalase serves as the primary H_2_O_2_-scavenging enzyme. Aging is associated with reduced catalase activity and mislocalization to the cytosol, compromising peroxisomal capacity to neutralize H_2_O_2_ and maintain redox balance [68]. Concurrent age-related impairments in pexophagy may lead to the accumulation of redox-compromised peroxisomes, further exacerbating functional decline. Because H_2_O_2_ can permeate across the peroxisomal membrane [69], excess H_2_O_2_ may spill into the cytosol, modulating H_2_O_2_-responsive signaling pathways at low concentrations or promoting oxidative stress at higher levels (Figure 1) [70]. Chronic pharmacological inhibition of catalase has been shown to induce cellular senescence, consistent with aging-associated phenotypes [32]. Despite these insights, many mechanistic details of how peroxisomal redox imbalance affects H_2_O_2_-mediated signaling and oxidative stress pathways remain to be elucidated.

### 3.4. Peroxisomal Lipid Metabolism

Peroxisomes are essential for the β-oxidation of specific fatty acids—including VLCFAs, pristanic acid, PUFAs, and bile acid intermediates—as well as for the biosynthesis of DHA and plasmalogens [13,14]. Many peroxisomal functions decline with age, including β-oxidation [71], plasmalogen biosynthesis [72], bile acid metabolism [52,73], and DHA synthesis [74] (Figure 1). Because these lipids and lipid intermediates play key roles in cellular signaling, membrane composition, and overall homeostasis, their dysregulation may contribute to age-associated cellular dysfunction [75]. Indeed, alterations in membrane phospholipids, PUFAs, plasmalogens, and DHA-containing or DHA-derived lipid mediators are correlated with age-related functional declines, including cognitive impairment, metabolic disturbances, and reduced lifespan [76]. Nevertheless, while these correlations are suggestive, direct causal links between peroxisomal lipid metabolism and lifespan in long-lived mammals and humans remain to be firmly established.

## 4. Peroxisomal Decline and Its Contribution to Age-Associated Pathologies

**Figure 2:**
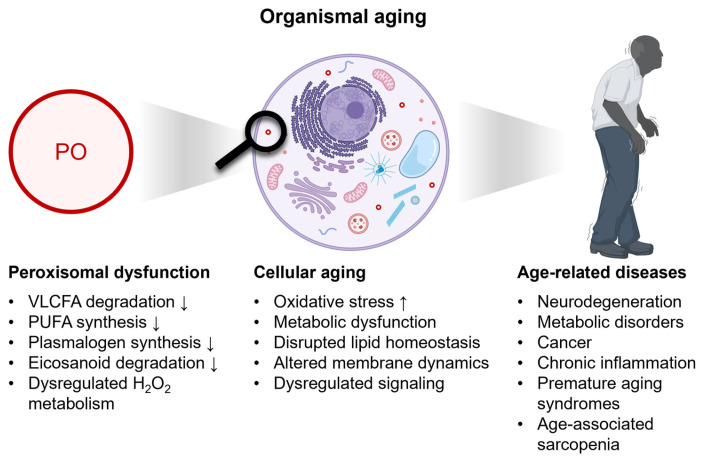
Peroxisomal dysfunction contributes to hallmarks of cellular aging and is associated with age-related diseases. Some elements were adapted from BioRender (https://www.biorender.com; accessed on 22 December 2025) templates. The magnifying glass highlights an individual peroxisome. ↑ indicates an increase; ↓ indicates a decrease; For details, see the main text.

Peroxisomal metabolism, particularly fatty acid β-oxidation and H_2_O_2_ metabolism, may be modulated by lifestyle factors such as high-fat diets, smoking, alcohol consumption, and physical activity. Consistent with this possibility, several studies have reported associations between these exposures and changes in peroxisomal gene expression or catalase activity [77,78]; however, most available evidence is indirect or derived from animal models [14,79,80,81,82,83], and causal effects in humans remain uncertain. Nevertheless, it is well established that a decline in peroxisomal function can lead to lipid accumulation, alterations in membrane composition, impaired antioxidant defenses, metabolic dysfunction, and disrupted cellular signaling, all of which contribute to cellular aging and age-related pathologies (Figure 2) [76]. Decreased plasmalogen levels can compromise antioxidant protection and membrane stabilization, while DHA supports neuronal health by maintaining membrane fluidity, promoting synaptic function, and serving as a precursor for neuroprotectin D1, a mediator with anti-inflammatory and neuroprotective effects. Deficits in these pathways have been associated with cognitive decline. This section reviews current insights into the role of peroxisomes in various age-related diseases.

### 4.1. Neurodegenerative Disorders

Age-related neurodegenerative disorders, including Alzheimer’s disease (AD), Parkinson’s disease (PD), and amyotrophic lateral sclerosis (ALS), are characterized by chronic oxidative stress, neuroinflammation, and progressive disturbances in peroxisomal metabolism [84,85,86,87]. Plasmalogens, ether phospholipids whose biosynthesis is initiated in peroxisomes and completed in the endoplasmic reticulum, are highly enriched in neuronal membranes, where they contribute to membrane organization and dynamics, exert endogenous antioxidant activity, and support processes essential for synaptic integrity and neuronal function [88,89]. Consistent with these roles, plasmalogen deficiency is increasingly recognized as a common feature of neurodegenerative disorders and is thought to contribute to disease pathogenesis [90,91].

In AD, postmortem analyses have revealed accumulation of VLCFAs, reduced plasmalogen levels, increased oxidative stress, and region-specific, disease stage-dependent alterations in peroxisome density. Notably, peroxisomes are selectively lost from neurites of neurons containing abnormally phosphorylated tau, whereas neuronal somata in advanced stages of AD exhibit increased peroxisome volume density compared with less affected individuals [19,92]. Lipidomic studies have demonstrated marked reductions in plasmalogen that correlate with synaptic dysfunction, cognitive decline, and neuropathological staging in AD, supporting a contributory—and potentially causative—role in disease progression [19,88,93].

In PD, lipid composition analyses of frontal cortical lipid rafts reveal marked reductions in DHA compared with control brains [94]. PD is also associated with impaired peroxisomal β-oxidation and reduced plasmalogen levels, and experimental studies indicate that pharmacological stimulation of peroxisome proliferation can mitigate α-synuclein-associated toxicity [95]. Although direct causal evidence linking plasmalogen depletion to α-synuclein aggregation remains limited, restoration of plasmalogen levels using plasmalogen precursors protects dopaminergic neurons from toxin-induced degeneration in PD models [96], consistent with plasmalogen deficiency contributing to increased dopaminergic vulnerability. In addition, supplementation with plasmalogens or derivatives may improve cognitive functions in patients with mild AD and enhance spatial memory while reducing neuroinflammation in mice [97,98]. More broadly, alterations in neuronal lipid composition, including specific phospholipid classes, are known to modulate α-synuclein aggregation dynamics and pathogenicity [99]. Furthermore, DHA has demonstrated neuroprotective effects in multiple PD models [100,101,102].

In ALS, decreased levels of DHA-related β-oxidation enzymes have been reported [103], along with reduced expression of alkylglycerone-phosphate synthase, a key peroxisomal enzyme required for plasmalogen biosynthesis, in motor neuron disease models [104]. Mutations in the peroxisomal enzyme D-amino acid oxidase have also been linked to ALS, contributing to peroxisomal dysfunction and oxidative damage [105].

Collectively, these observations support the emerging concept that age-associated peroxisomal dysfunction disrupts peroxisomal lipid metabolism, including plasmalogens, representing a central metabolic alteration that contributes to neuronal vulnerability and disease progression across multiple neurodegenerative disorders.

### 4.2. Metabolic Disorders

Peroxisomes are central regulators of cellular lipid metabolism, oxidizing very-long-chain and complex fatty acids, generating signaling metabolites such as acetyl-CoA and H_2_O_2_, and synthesizing ether lipids [106,107]. Through these activities, they contribute to whole-body energy homeostasis, brown adipose tissue thermogenesis, lipolysis, lipid droplet turnover, and metabolic signaling [108,109]. Peroxisomes also engage in dynamic interactions with mitochondria, the ER, lipid droplets, and lysosomes, enabling coordinated lipid metabolism and interorganelle metabolite exchange [22].

The essential role of peroxisomes in human physiology is underscored by genetic disorders caused by defects in peroxisome biogenesis or enzyme function. These inborn errors of metabolism are characterized by severe multi-organ pathology, neurological impairment, and early mortality. Examples include Zellweger spectrum disorders, X-linked adrenoleukodystrophy, Refsum disease, and acyl-CoA oxidase deficiency [28,110]. Beyond these rare conditions, human and animal studies demonstrate that peroxisomes are critical regulators of obesity, dyslipidemia, and systemic glucose homeostasis [109,111,112].

In metabolic disease, peroxisomal dysfunction contributes directly to tissue-specific pathology. In nonalcoholic fatty liver disease (NAFLD), impaired peroxisomal metabolism promotes hepatic lipid accumulation and oxidative stress [79]. In obesity and hyperlipidemia, enhanced peroxisomal β-oxidation increases H_2_O_2_ production, contributing to pancreatic β-cell lipotoxicity and impaired insulin secretion, while reduced plasmalogen levels and altered ether lipid composition exacerbate diabetic neuropathy and other metabolic complications [108,113,114]. Accordingly, intact peroxisomal function is essential for β-cell integrity and insulin secretion [115]. Diabetes is further associated with impaired peroxisome biogenesis, resulting in increased ROS levels, blunted insulin secretion, and aggravated lipotoxicity [116]. Consistently, catalase deficiency accelerates diabetic renal injury by compromising peroxisomal redox homeostasis [117]. In white adipose tissue, obesity is linked to reduced expression of peroxisomal genes and diminished peroxisomal fitness, fostering oxidative stress and inflammation [112]. In line with these findings, lower circulating plasmalogen levels correlate with higher body mass index and increased metabolic risk [118,119].

Age-related disruption of peroxisomal function and interorganelle communication further exacerbates metabolic stress by impairing lipid mobilization, fatty acid metabolism, contact site-mediated metabolite transfer, and ER stress responses [29,120,121]. These alterations reduce metabolic flexibility, limiting the ability to switch energy substrates during fasting or dietary interventions [122]. With advancing age, changes in peroxisome biogenesis, enzyme activity, and signaling drive a progressive loss of metabolic flexibility—a hallmark of aging and a shared feature of metabolic syndrome and type 2 diabetes [123,124]—thereby increasing susceptibility to insulin resistance and hepatic steatosis [109,122]. Consequently, peroxisomal dysfunction is increasingly recognized as a central contributor to age-related metabolic diseases, including type 2 diabetes and its complications, NAFLD, metabolic syndrome, dyslipidemia, and obesity [29]. Beyond metabolic tissues, peroxisomal impairment in the nervous system is also increasingly implicated in neurodegeneration, where disrupted lipid homeostasis, defective myelin maintenance, and elevated oxidative stress comprise neuronal integrity [125].

### 4.3. Cancer

Peroxisomes exhibit a context-dependent, Janus-faced role in cancer, functioning either as tumor suppressors or as metabolic enablers depending on cellular state and disease stage [126]. Under homeostatic conditions, peroxisomes maintain redox and lipid balance through catalase-mediated detoxification and fatty acid metabolism, thereby preventing oxidative stress, genomic instability, and lipotoxicity. During aging, peroxisomal dysfunction can contribute to cellular senescence and the formation of a pro-tumorigenic microenvironment. In senescent cells, reduced peroxisomal catalase increases ROS levels, and altered lipid metabolism impairs membrane properties, both of which affect intracellular signaling [29,32,127,128,129]. Concurrently, impaired pexophagy leads to the accumulation of dysfunctional peroxisomes [66], further reducing metabolic flexibility, elevating oxidative stress, and causing the buildup of VLCFAs, collectively promoting chronic low-grade inflammation and facilitating cancer initiation [129,130,131,132]. Supporting this, reduced catalase expression correlates with poor prognosis in several cancers, partly due to enhanced ROS-driven oncogenic signaling [133,134].

Conversely, once tumors are established, peroxisomes are frequently co-opted to support cancer progression [135,136,137]. Peroxisomal β-oxidation supplies acetyl-CoA for lipid biosynthesis, H_2_O_2_ functions as a pro-tumorigenic signaling molecule, and plasmalogens enhance membrane dynamics, cell survival, and metastatic potential. Thus, while age-associated peroxisomal dysfunction initially promotes cellular senescence, selective reactivation and metabolic rewiring of peroxisomes during tumor initiation and progression support metabolic plasticity and contribute to malignant progression.

### 4.4. Inflammatory-Related Disorders

Peroxisomes play a critical role in modulating inflammation through lipid metabolism, redox homeostasis, and signaling pathways [130]. They degrade pro-inflammatory eicosanoids, including prostaglandins, thromboxanes, leukotrienes, and prostacyclins [138], while also contributing to the synthesis of PUFAs such as DHA, which are precursors of anti-inflammatory mediators including resolvins, maresins, and protectins [139,140]. Oxidative stress and inflammation mutually reinforce one another [131], modulating key inflammatory pathways such as NF-κB and MAPK [130].

Peroxisomal dysfunction disrupts lipid metabolism and redox homeostasis, leading to oxidative stress and dysregulated inflammatory responses [131]. For example, reduced peroxisomal β-oxidation causes accumulation of VLCFAs, which can promote the production of inflammatory mediators [14,132]. VLCFA buildup can further impair peroxisomal function, creating a self-amplifying cycle of inflammation [27,141].

An example of an age-related inflammatory pathology is multiple sclerosis (MS), a chronic autoimmune disease of the central nervous system characterized by neuroinflammation, demyelination, and axonal loss [142]. Although the mechanisms underlying MS are not fully understood, peroxisomes have been implicated in its pathogenesis [143]. In animal models, peroxisome deficiency in neural cells induces rapid neuroinflammation [144]. Experimental MS models exhibit reduced catalase activity, decreased plasmalogen levels, and VLCFA accumulation, which may increase tumor necrosis factor-alpha production and drive demyelination [145]. Consistently, tissues from MS patients show reduced neuronal peroxisome abundance alongside elevated VLCFA levels [146,147].

### 4.5. Premature Aging Syndromes

Progeroid syndromes exhibit features resembling normal aging, but these manifest earlier in life and progress rapidly. As such, they provide an accelerated model for studying aging mechanisms [148]. These syndromes can be broadly classified into two groups: (i) laminopathies, arising from defects in the nuclear lamina, and (ii) syndromes caused by defects in DNA repair or the DNA damage response [149,150].

Hutchinson-Gilford progeria syndrome (HGPS) is a laminopathy caused by mutations in the *LMNA* gene, leading to accumulation of progerin, a truncated form of the Lamin A protein. HGPS patients display accelerated aging phenotypes, including growth retardation, fat loss, hair thinning, bone erosion, and skin tightening, often resulting in early death due to cardiovascular complications [151]. Similarly to normal aging, HGPS is characterized by elevated ROS, stemming from mitochondrial dysfunction [152]. While peroxisome density remains unchanged in HGPS cells, antioxidant defenses are affected: catalase activity is significantly reduced [152], whereas glutathione peroxidase shows variable effects—decreasing in some studies [153] but unaffected in others [152]. HGPS cells also exhibit impaired protein import and reduced PEX5 expression, likely exacerbating oxidative stress and promoting cellular senescence [152].

Ataxia-telangiectasia (A-T), a representative example of the second group of progeroid syndromes, is caused by mutations in the *ATM* gene, which impair DNA break repair and result in genomic instability, cerebellar degeneration, immune deficiencies, increased cancer risk, and radiation hypersensitivity [150,154]. Under oxidative stress, ATM phosphorylates PEX5, promoting its ubiquitylation at the peroxisomal membrane and recognition by autophagy receptor p62, while simultaneously inhibiting mTORC1 to stimulate pexophagy [155]. In A-T patients, loss of functional ATM prevents proper mTORC1 repression [156], which may impair pexophagy and thereby contribute to elevated oxidative stress and cellular senescence.

### 4.6. Age-Associated Sarcopenia

Age-related sarcopenia is a progressive muscle disease that develops with aging and is characterized by the loss of muscle mass, strength, and function [157]. This condition increases the risk of falls, fractures, disability, and reduces quality of life [158].

A recent study reported that peroxisomes are essential for efficient lipid metabolism in muscle cells, and that peroxisomal dysfunction impairs muscle energy production and redox balance [25]. Muscle-specific deletion of PEX5 in mice caused early disruption in lipid and amino acid metabolism, resulting in reduced muscle force, poor exercise performance, and progressive mitochondrial degeneration. These alterations were accompanied by sarcomere and neuromuscular junction deterioration, protein aggregate accumulation, and muscle atrophy, leading to premature muscle aging. The study also demonstrated that peroxisomal content in muscle declines progressively with age, emphasizing the importance of maintaining peroxisomal function and its influence on mitochondrial fitness to preserve muscle health during aging [25].

## 5. Targeting Peroxisomes to Promote Healthy Aging

### 5.1. Peroxisomes as Active Regulators of Cellular Aging

Peroxisomes are increasingly recognized as active regulators of cellular aging rather than passive metabolic components [26]. Age-associated declines in peroxisome abundance and function contribute to lipid dysregulation and elevated oxidative stress—hallmarks strongly associated with functional decline and age-related disease [159]. Beyond their intrinsic metabolic roles, peroxisomes engage in extensive crosstalk with other aging-relevant organelles, particularly mitochondria, through shared lipid metabolic pathways and redox signaling [21]. Peroxisomal dysfunction can therefore compromise mitochondrial integrity and bioenergetic capacity, amplifying cellular stress and accelerating aging phenotypes [160,161,162]. Through these interactions, peroxisomes influence broader metabolic and stress-response pathways that are central to longevity regulation.

Genetic and metabolic studies have further demonstrated that peroxisomal homeostasis directly influences aging and lifespan. In *C. elegans*, lifespan extension from dietary restriction and AMPK signaling depends on mitochondrial network remodeling and communication with peroxisomes, highlighting peroxisomal involvement as a downstream mechanism of these interventions [163]. Similarly, in *Drosophila*, age-dependent impairment of peroxisomal protein import in oenocytes activates c-Jun N-terminal kinase (JNK) signaling and increases the pro-inflammatory cytokine unpaired 3 (Upd3) [49]. Upd3 acts as a circulating factor that non-autonomously drives cardiac aging, linking peroxisomal decline to systemic aging processes [49].

Additional studies in *C. elegans* support a pro-longevity role for peroxisomes through their regulation of lipid metabolism and redox balance. Dietary mono-unsaturated fatty acids (MUFAs) increase both lipid droplet and peroxisome abundance in fat storage tissues, and this coordinated upregulation is required for MUFA-induced lifespan extension [164]. Similarly, dietary supplementation with α-ketobutyrate extends lifespan by elevating NAD^+^ levels via lactate dehydrogenase 1 [165]. Increased NAD^+^ activates SIR-2.1, the worm homolog of SIRT1, which promotes peroxisome biogenesis and fatty acid β-oxidation. This metabolic shift enhances H_2_O_2_ production, activating the SKN-1/Nrf2 stress response pathway and thereby stimulating antioxidant defenses, autophagy, and mitochondrial function to promote longevity [165]. Moreover, peroxisomal ether lipid biosynthesis is required for lifespan extension across multiple pro-longevity paradigms, including treatment with the biguanide drug phenformin, a metformin analog [166]. Consistently, peroxisomal protein import, fatty acid elongases, and desaturases are essential for the longevity-promoting effects of biguanides, underscoring the critical role of peroxisomal lipid metabolism in aging regulation [166].

Collectively, these findings position peroxisomes as central regulators of aging, whose metabolic and signaling functions integrate with those of other organelles to influence organismal longevity.

### 5.2. Crosstalk Between Peroxisomes and Longevity-Associated Signaling Pathways

Peroxisomes intersect with several key longevity-associated signaling pathways, including AMPK/SIRT1/PGC-1α, mTOR, Nrf2, and FOXO3 [167,168,169,170,171]. Through these signaling axes, they contribute to the coordination of metabolic adaptations, stress resistance, and redox homeostasis. For example, activation of Nrf2 enhances the expression of catalase, a major peroxisomal antioxidant enzyme, thereby mitigating oxidative damage and preserving redox homeostasis [172]. Maintenance of peroxisomal health may therefore support cellular homeostasis, preserve mitochondrial function, and counteract age-related dysfunction across multiple tissues [173].

### 5.3. Preserving Peroxisomal Homeostasis During Aging

**Figure 3:**
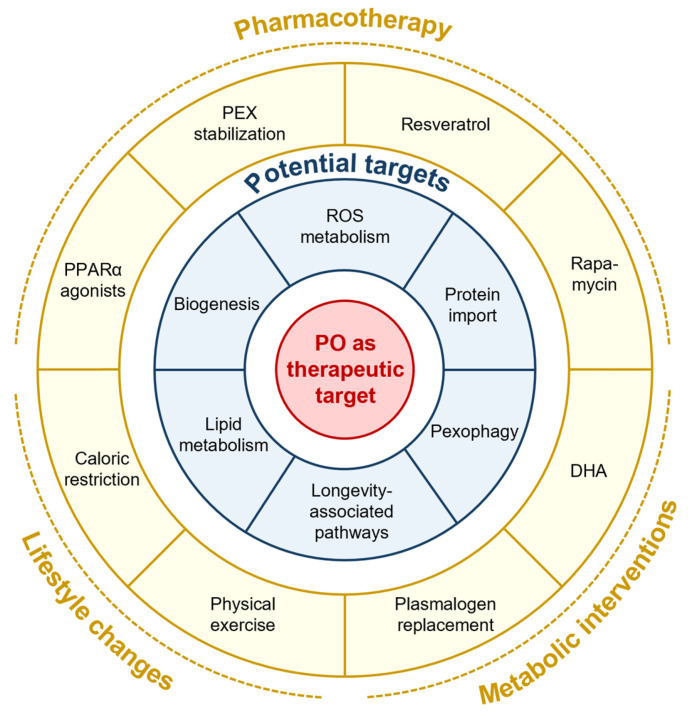
Potential interventions to preserve peroxisome function during cellular and organismal aging. For details, see the main text.

Strategies aimed at preserving or enhancing peroxisomal function during aging include promoting peroxisome biogenesis and protein import, fine-tuning selective autophagy of damaged peroxisomes, and maintaining balanced ROS signaling (Figure 3). Sustaining the molecular machinery responsible for peroxisome formation and enzyme import can help maintain organelle number and functionality with age. Likewise, appropriate regulation of pexophagy prevents the accumulation of dysfunctional peroxisomes, although both excessive and insufficient peroxisome turnover may have context-dependent deleterious effects [174,175]. Given the central role of peroxisomes in ROS metabolism, interventions that support ROS hormesis—preserving beneficial signaling while limiting oxidative damage—may be particularly effective in maintaining cellular function during aging.

### 5.4. Pharmacological Modulation of Peroxisomal Pathways

Pharmacological interventions targeting peroxisomal pathways have shown promise in preclinical models [176]. Activation of peroxisome proliferator-activated receptors (PPARs), particularly PPARα, can restore peroxisomal activity, enhance fatty acid oxidation, and protect against age-associated metabolic disorders, including NAFLD, dyslipidemia, metabolic syndrome, and type 2 diabetes, in aging models [177,178,179,180,181,182,183]. However, the pleiotropic effects of PPAR signaling extend beyond peroxisome regulation and therefore warrant careful consideration [180].

Modulation of peroxisome biogenesis factors also represents a complementary strategy to enhance peroxisome abundance. For example, DHA induces peroxisome elongation through PEX11B oligomerization, a process that is prerequisite for peroxisome division [184]. In addition, certain small molecules and ligands can stabilize mutant PEX1, PEX6, or PEX12, thereby partially restoring peroxisomal matrix protein import, while defects in plasmalogen biosynthesis may be bypassed through plasmalogen replacement [185,186,187,188]. Collectively, these approaches alleviate cellular stress by restoring or circumventing key peroxisomal metabolic functions, including fatty acid β-oxidation and plasmalogen biosynthesis. Moreover, activation of the AMPK/SIRT1/PGC-1α signaling pathway, either pharmacologically or through caloric restriction, is likely to enhance peroxisome biogenesis, stimulate fatty acid oxidation, and limit the accumulation of damaged peroxisomes, thereby reinforcing longevity-associated stress-response pathways [170,189].

### 5.5. Lifestyle Interventions and Systemic Modulators of Peroxisomal Function

Lifestyle interventions and pharmacological agents—including caloric restriction, physical exercise, and compounds such as fibrate drugs, resveratrol, and rapamycin—have demonstrated, either directly or indirectly, potential to restore peroxisomal homeostasis and support healthy aging in model systems [79,80,170,190,191,192,193,194,195,196,197,198]. These interventions may fine-tune peroxisome abundance, redox balance, and organelle turnover by engaging conserved nutrient- and energy-sensing pathways. Nevertheless, given the pleiotropic nature of many small-molecule interventions, potential off-target effects and context-dependent outcomes require careful evaluation.

### 5.6. Future Directions: Peroxisome-Targeted Strategies for Healthy Aging

Overall, targeting peroxisomes represents a promising yet still largely experimental strategy to promote healthy aging. By preserving lipid and ROS metabolism, maintaining mitochondrial health, and modulating conserved longevity pathways, peroxisome-targeted interventions may mitigate age-associated metabolic and neurodegenerative disorders. While current evidence is predominantly derived from preclinical studies, future research should determine whether peroxisome-targeted strategies can meaningfully improve healthspan and lifespan in humans.

## 6. Conclusions

Peroxisomes are increasingly recognized as underappreciated regulators of aging that influence cellular resilience and systemic metabolism. As peroxisomal dysfunction has been implicated in various age-related disorders, a deeper understanding of peroxisomal biology—including organelle biogenesis, metabolic and signaling functions, and pexophagy—is essential to elucidate how these organelles safeguard healthy aging and contribute to age-associated disease. Although targeting peroxisomes represents a promising therapeutic avenue, strategies aimed at modulating peroxisomal function as anti-aging interventions remain in their infancy, underscoring the need for further mechanistic and translational research.

## Data Availability

All information in this review is derived from published literature cited in the manuscript; no new data were generated or analyzed, and data sharing is therefore not applicable.

## Abbreviations

The following abbreviations are used in this manuscript:

AD	Alzheimer’s disease
ALS	Amyotrophic lateral sclerosis
AMPK	5′-AMP-activated protein kinase catalytic subunit alpha-1
A-T	Ataxia-telangiectasia
DHA	Docosahexaenoic acid
ER	Endoplasmic reticulum
HGPS	Hutchinson-Gilford progeria syndrome
MS	Multiple sclerosis
mTOR	Mammalian target of rapamycin
MUFA	Mono-unsaturated fatty acid
NAFLD	Nonalcoholic fatty liver disease
PD	Parkinson’s disease
PEX	Peroxin
PMP	Peroxisomal membrane protein
PPAR	Peroxisome proliferator-activated receptor
PTS	Peroxisomal targeting signal
PUFA	Poly-unsaturated fatty acid
ROS	Reactive oxygen species
SIRT1	NAD-dependent protein deacetylase sirtuin-1
VLCFA	Very-long-chain fatty acid
